# Enhancement of Tumorigenicity, Spheroid Niche, and Drug Resistance of Pancreatic Cancer Cells in Three-Dimensional Culture System

**DOI:** 10.7150/jca.87494

**Published:** 2024-03-02

**Authors:** Hao-Chien Hung, Tsui-Lien Mao, Ming-Huei Fan, Guan-Zhi Huang, Ainani Priza Minhalina, Chi-Long Chen, Chao-Lien Liu

**Affiliations:** 1 Department of General Surgery, Chang-Gung Memorial Hospital at Linkou, Taoyuan 33305, Taiwan.; 2 Department of Pathology, College of Medicine, National Taiwan University, Taipei 10002, Taiwan.; 3 Department of Pathology, National Taiwan University Hospital, Taipei 10002, Taiwan.; 4 School of Medical Laboratory Science and Biotechnology, College of Medical Science and Technology, Taipei Medical University, Taipei 11031, Taiwan.; 5 Department of Pathology, School of Medicine, College of Medicine, Taipei Medical University, Taipei 11031, Taiwan.; 6 Department of Pathology, Taipei Medical University Hospital, Taipei 11031, Taiwan.; 7 PhD Program in Medical Biotechnology, College of Medical Science and Technology, Taipei Medical University, Taipei 11031, Taiwan.

**Keywords:** Pancreatic cancer (PaC), cancer-associated fibroblast (CAF), two dimensional (2D), three dimensional (3D), spheroid model, CXCR3/CXCL10 axis, therapeutic discovery.

## Abstract

The three-dimensional (3D) cell culture technique has been applied comprehensively as a variable platform for medical research, biochemical signal pathway analysis, and evaluation of anti-tumor treatment response due to an excellent recapitulation of a tumor microenvironment (TME) in the *in vitro* cultured cancer cells. Pancreatic cancer (PaC) is one of the toughest malignancies with a complex TME and refractory treatment response. To comprehensively study the TME of PaC, there is an eager need to develop a 3D culture model to decompose the cellular components and their cross interactions. Herein, we establish a 3D PaC culture system with cancer stem cell (CSC) and scalability properties. To validate our model, we tested the individual PaC cell and the combined effects with cancer-associated fibroblasts (CAFs) on cancer tumorigenicity, the cellular interaction through the CXCR3/CXCL10 axis, and cellular responses reflection of anti-cancer treatments. With the help of our 3D technology, a simulated malignant spheroid with important stromal populations and TME physiochemical properties may be successfully recreated. It can be used in a wide range of preclinical research and helpful in advancing basic and translational cancer biology.

## Introduction

Pancreatic cancer (PaC) is an aggressive malignancy with inferior survival despite novel therapies, such as immunotherapy in combination with conventional chemotherapy and radiotherapy^1^-^3^. The tumor microenvironment (TME) is an intricate and dynamic niche encompassing tumor cells, immune cells, stromal cells, and cancer-associated fibroblasts (CAFs), collaborating in regulating cancer progression, response to therapies, and development of chemoresistance 4, 5. Currently, the treatment of PaC remains a significant challenge despite improved understanding through ever-evolving biotechnology. The aggressive nature of the disease and the lack of efficacious therapy have driven the development of an *in vitro* tumor model to deconstruct the TME components and epitomize the complexity of tumor biology 6, 7. Compared to traditional two-dimensional (2D) monolayer, a three-dimensional (3D) culture system is more competent in recapitulating the TME 8-12. In addition, the 3D culture model is more cost-saving, has faster throughput, and is more capable of being fully controlled and easily reproduced than a living animal model, particularly in cancer-related research 13, 14. Furthermore, the co-culture of cancer cells with CAFs has been regarded as an effective method for recreating functional TME *in vitro* 15.

Advantages in the utility of organoids and patient-derived cancer cell models are irreplaceable. However, a significant disparity with the original cancer cells makes it almost impossible to replicate identical cultures 16. As PaC tumor biology and its TME is better understood; efforts have been made to disclose cellular pathways that contribute to tumor invasiveness, discover novel therapies, and conduct therapeutic screening, all of which require a large number of high-quality cells 17, 18. Among these, induced pluripotent stem cells (iPSCs) are a suitable solution because of their exceptional ability for boundless *in vitro* expansion 19. As stated above, there is an urgent need to develop a simply constructed and reliable 3D iPSC PaC culture system that can accomplish pre-clinical exploration of tumorigenicity, tumor biology, treatment response, and therapeutic discovery.

In this study, we developed an easily reproducible cancer stem cell (CSC)-like PaC model using 3D culture conditions that exhibit enhanced tumorigenicity and invasiveness. A representative interaction between CXCR3 expressed by activated T cells and CXCL10 secreted by PaC cells was explored. This PaC spheroid in a 3D model can be used as a chemoresistant chimeric antigen receptor (CAR)-T cell therapeutic platform to validate preclinical effects.

## Materials and Methods

### Cell Lines and Two-Dimensional (2D)-Monolayer Cell Culture

Human PaC cell lines (CFPAC1, SU8686, HPAF2, ASPC1, MIAPaCa-2, Capan1) were purchased from AmericanType Culture Collection (ATCC, Manassas, VA, USA), while primary human cancer-associated fibroblast (CAF) was purchased from Neuromics (A division of CA3 Biosciences, Inc., Edina, MN, USA). All cells were maintained in Dulbecco's modified Eagle'smedium (DMEM; Life Technologies, Carlsbad, CA, USA) supplemented with 10% fetal bovine serum (FBS; HyClone, Logan, UT, USA) at 37º C with 5% CO_2_ incubation. All cell lines were tested to identify the species and detect mycoplasma, and their original manufacturers confirmed authentication using a short-tandem repeat (STR) profile analysis.

### *In Vitro* Overexpression of Green Fluorescent Protein (GFP)

The pRRLMNDkGFP lentiviral vector was purchased from Addgene (Watertown, MA, USA), and all plasmid DNA was purified using an Endo-Free Maxi prep kit (Qiagen, Valencia, CA, USA). Briefly, human 293T cells were seeded at 9 × 10^6^ cells/15-cm dish 24 h before transfection. Viral particles were then generated with 7.5 μg of pRRLMNDkGFP plasmid vector in addition of 4.5 μg psPAX2 packaging plasmid and 3 μg pMD2G envelope plasmid using a calcium phosphate transfection system 20. Viral supernatants were harvested at 48 h after transfection. GFP was overexpressed in PaC cell lines (MIA PaCa-2 and Capan1) and CAFs. All three cell types were individually seeded at a density of 5 × 10^5^ cells/well on a 24-well plate. 2 mL of fresh media containing 8 μg/mL polybrene (Sigma-Aldrich, St. Louis, MO, USA) and the recombinant lentivirus was added. All three MIA PaCa-2, Capan1, and CAF cells with stable GFP overexpression were used for subsequent assays following transduction and verification.

### SphereFormation Assay

For the sphere-formation assays, cells were harvested and resuspended in a defined serum-free medium (DMEM/F12) (Corning, Corning, NY, USA) containing 0.3% bovine serum albumin (Sigma-Aldrich, St. Louis, MO, USA), 5 μg/mL insulin (Thermo Fisher Scientific, Carlsbad, CA, USA), 10 ng/mL epidermal growth factor (EGF) (Thermo Fisher Scientific), 10 ng/mL essential fibroblast growth factor (β-FGF) (ThermoFisher Scientific), and 12 ng/mL leukemia inhibitory factor (LIF) (Thermo Fisher Scientific), and plated in triplicates into 1% chitosan (Sigma-Aldrich)-precoated 6-well plates (Corning) at a density of 2 × 10^5^cells/well. Cultural media were replaced every three days during the sphere induction period. Sphere cells were counted under an Olympus BX50 light microscope (Tokyo, Japan) at each indicated time point.

### Standard Three-Dimensional (3D) Cultures

To set up a 3D culture system, PaC and CAF cells (5 × 10^4^ cells/each line) were individually- or co-seeded in 24-well plates and grown in Matrigel (Corning Incorporated, Tewksbury, MA, USA). Briefly, cells were mixed with culture media; Matrigel was thawed overnight and poured into 24-well (the mixture of Matrigel to culture media ratio at 1) plates on ice. Matrigel was incubated for 30 min at 37ºC for polymerization and then covered by culture media. The culture media was replaced every two days; all cells were routinely cultured in a humidified atmosphere with 5% CO_2_ at 37ºC.

### Flow Cytometry

Cancer cell lines and spheroid cells were collected and prepared in triplicates. The following monoclonal antibodies were obtained from BD Biosciences (Los Angeles, CA, USA): phycoerythrin (PE) mouse anti-human cluster of differentiation (CD) 44, allophycocyanine (APC) mouse anti-human CD133, Alexa 647 mouse anti-human CD324 (E-Cadherin), PE mouse anti-human CD325 (N-Cadherin), and PE mouse anti-human CD183 (CXCR3) and their immunoglobulin G (IgG) controls. Secreted human CXCL10 were measured by human IP-10 Flex Set [BD cytometric bead array (CBA)]. Reacted cells or culture media were analyzed using an AttuneTM NxT Acoustic Focusing Cytometer (Thermo Fisher Scientific). AttuneTM NxT software (Thermo Fisher Scientific) was used for measurement and data processing of each flow cytometry analysis.

### Real-Time Reverse-Transcription Quantitative Polymerase Chain Reaction (RT-qPCR)

Cells were collected and lysed for total RNA extraction using the LabPrep RNA Plus Mini Kit (Taigen Bioscience). Complementary DNA (cDNA) was synthesized by reverse-transcription of RNA using High-Capacity cDNA Reverse Transcription Kits (Thermo Fisher Scientific) according to our previous work 21. Briefly, the qPCR was performed in triplicate for each cDNA sample on an Applied Biosystems StepOne and StepOnePlus Real-Time PCR System (Applied Biosystems; Thermo Fisher Scientific) using PowerUp SYBR Green Master Mix (Thermo Fisher Scientific). The crossing threshold (Ct) value of the transcripts assessed by the RT-qPCR was normalized to that of the human S26 gene. Changes in messenger (m)RNA levels are expressed as multiples of change relative to the control ± SD. The primer sequences used are listed below.

### Drug-Resistance and Cell Viability Assay

A3-(4,5-dimethylthiazol-2-yl)-2,5-diphenyltetrazolium bromide (MTT; Thermo Fisher Scientific) assay was used for the determination of cell viability as previously described 21. Briefly, cells at 3 × 10^4^ cells/well were triplicated and seeded into 96-well plates, cultured with complete medium for 16 h, and then treated with different regimens including CAR-T cells, cisplatin (CDDP) (Sigma-Aldrich), paclitaxel (PTX) (Sigma-Aldrich), or their combination for specific time points. After removing the supernatant, DMEM containing 5 mg/mL MTT was added and incubated for four h. The resulting formazan was dissolved in 1 mL isopropanol, and the absorbance at 570 nm was measured using a microplate reader (Bio-Rad Laboratories, Hercules, CA, USA). The number of viable cells was proportional to the absorbance, and cell viability was presented as a percentage of the dimethylsulfoxide (DMSO) control (untreated).

### Transwell Invasion Assay

The invasion assay was performed using 24-well transwell chambers containing membranes coated with Matrigel (Merck Millipore, St. Louis, MO, USA). 3D cultured cells were seeded at 4 × 10^4^ cells/well in the upper chamber while RPMI 1640 containing 10% fetal bovine serum (FBS) was added to the lower chamber. Following 24 h of incubation, cells that had not migrated across the membrane were removed with a cotton swab, whereas cells that had migrated to the lower surface of the inserts were stained by diamidino-2-phenylindole (DAPI) for 10 min before the observation.

### T Cell Recruitment Assay

The T cell recruitment assay was performed using 24-well transwell chambers. Briefly, 3D cultured cells were seeded at 2 × 10^5^ cells/well into the lower chambers, while naïve or effector T cells at 4 × 10^4^ cells/well containing RPMI 1640 media were then seeded into the upper chambers. Following 30 h of incubation, T cells that had migrated to the lower surface of the inserts were stained by DAPI as previous described. T cells that had migrated into the lower chamber wells were also employed for cytotoxic activity examination toward tumor spheres by MTT assays.

### Statistical Analysis

All data arepresented as the mean ± SD of three independent experiments. A two-tailed *t* test was used for intergroup comparison. Comparisons between groups were determined with a one-way ANOVA analysis. Differences were considered significant at *p* < 0.05 using SPSS Statistics version 24.0 (SPSS, Inc., Chicago, IL, USA).

## Results

### Establishment of sphere-forming PaC and CAF cells with CSC properties

First, we assessed the stemness and proliferation activities of six human PaC cell lines (CFPAC1, SU8686, HPAF2, ASPC1, MIAPaCa-2, and Capan1) under a 2D culture condition for eight days (***Supplementary Figure 1***). The positive expression of the surface markers, CD44 and CD133, has been widely employed to recognize postulated CSCs 22. Morphologic and cell-fold changes observed by optical microscopy seemed to be positively correlated with CD44 and CD133 expression analyzed by flow cytometry during the sphere-induction period (***Supplementary Figure 1***).

**Figure 1:**
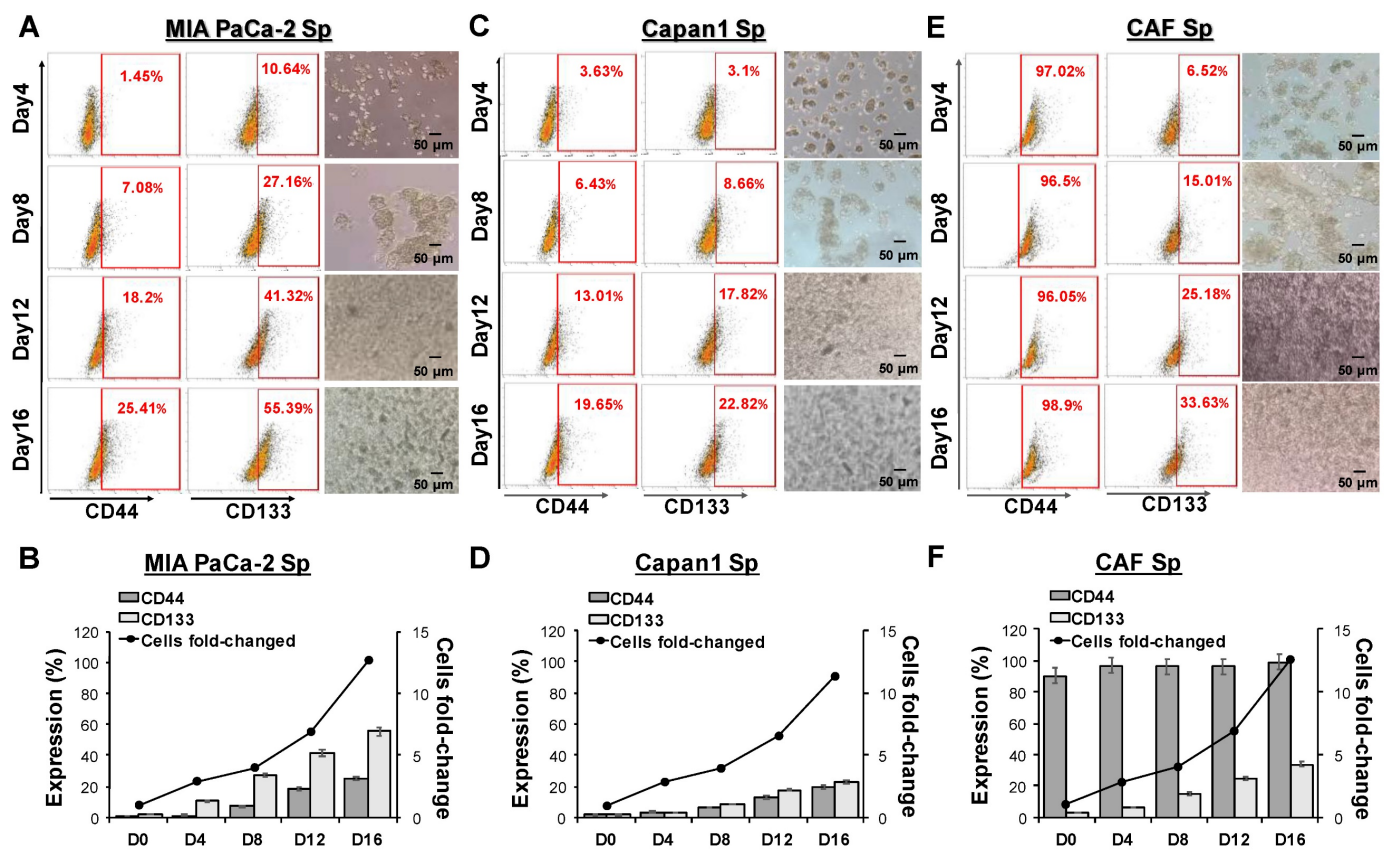
** Morphological changes and stemness markers expression of pancreatic cancer (PaC)- and cancer-associated fibroblast (CAF)-sphere cells in 3D culture.** PaC-sphere cells derived from human **(A)** MIA PaCa-2, **(B)** Capan1, and CAF-sphere cells derived from human **(C)** CAF cells, respectively at days 4, 8, 12, and 16 using 3D culture system. Stemness markers CD44 and CD133 in **(D)**MIA PaCa-2, **(E)** Capan1, and **(F)** CAF spheres were characterized by flow cytometry according to different cultured days. Morphological changes of formative spheres were directly observed by optical microscopic examination during the sphere-induction period. The magnitude of change in CD44 and CD133 expression, and cell-fold change values are displayed with respect to the different time points. Data presented are the mean ± SD of triplicate independent experiments. Scale bar = 50 μm.

To further establish the sphere-forming ability of the two selected PaC cells (MIA PaCa-2 and Capan1) and CAFs under a 3D culture condition employed, individual cell suspensions plated in ultra-low attachment plates were cultured in a sphere-induction defined medium for specific time points at days 4, 8, 12, and 16. Optical microscopy confirmed the sphere-forming capability of the adhering cells (***Figures 1A, 1C, and 1E***). CAF cells were formed into a spheroid structure (refer to CAF-spheres) under sphere-forming assays. Furthermore, the increase in cell fold-change indicated significant proliferative activity and reached 10 to 15 times their orginal numbers at day 16 (***Figure 1B, 1D, and 1F***), indicating that spheres increased over time following cultivation. To investigate whether 3D sphere cells acquire CSC properties, the stem cell markers CD44 and CD133 were analyzed by using flow cytometry analysis. After 16 days of culture, the number of CD44 and CD133 positive cells increased, with populations of 25.41% and 55.39%, 19.65% and 22.82%, 98.90% and 33.63% for MIA PaCa-2, Capan1, and CAF cells, respectively (***Figure 1A, 1C, and 1E***). A spheroid in a 3D culture setting is more effective and of better quality in terms of spheroid number, structure, size, and stemness features than in a 2D environment (***Supplementary Figure 2***).

The changes in morphology and stemness markers have been observed in the spheres of ovarian cancer cells (SKOV3) and normal human fibroblasts (WS1), which serve as the external controls to explore sphere formation and ability to express stemness characteristics, as shown in **Supplementary Figure 3**. A relatively small percentage of WS1 cells were CD44 (1.10%) and CD133 (1.80%) positive on day 3, and limited elevation on CD44 (10.10%) and CD133 (3.26%) on day 3. In contrast, the SKOV3 spheres express CD44 in high levels at each time points (92.70% and 90.35%, respectively), and an elevation on CD133 expression were observed (from 8.21% on day 3 to 14.92% on day 6). The cell fold change was checked on post incubation day 3 and day 6, and the WS1 and SKOV3 spheres achieved proportional growth between day 3 and day 6, and the latter cells seem to have robust proliferation.

**Figure 2:**
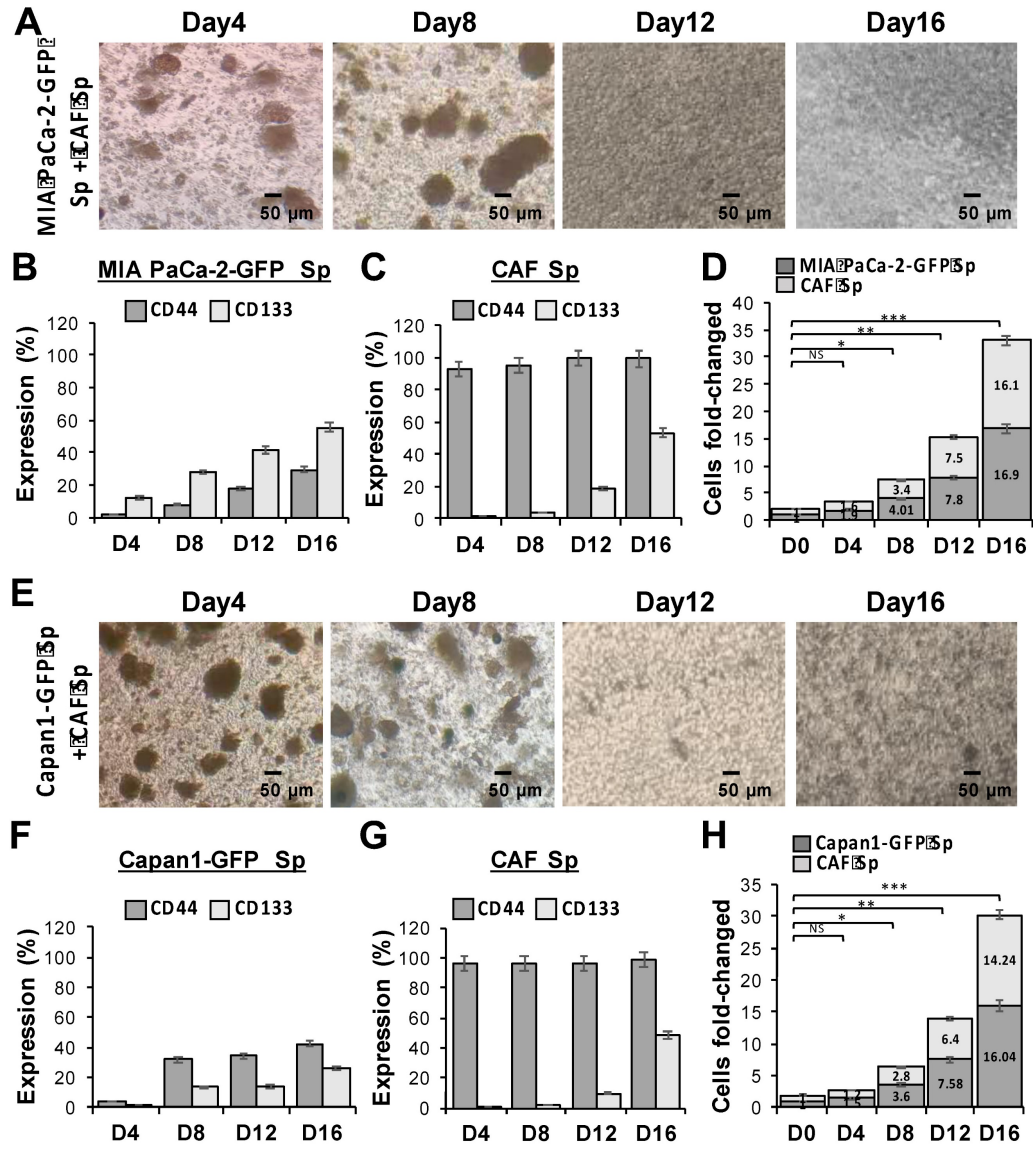
** Significantly increased cell proliferation and stemness markers expression in PaC cocultured with CAF using a 3D culture system.** Evaluation of multicellular 3D cultures of tumor spheres with cancer stem cell (CSC)-like cells markers. GFP-transduced human PaC cells, (A to D) MIA PaCa-2-GFP and (E to H) Capan1-GFP, were co-cultured with CAF cells (CAF), respectively during the sphere induction period. Optical microscopic images showed morphological changes of **(A)** MIA PaCa-2 + CAF and **(E)** Capan1+ CAF co-cultured at days 4, 8, 12, and 16. The magnitude of change in (B, C, and F, G) stemness markers CD44/CD133 expression and (D and H) significant cell fold change values of each cell line from a 3D co-cultured system are displayed concerning different time points (*p < 0.05; **p < 0.01; ***p < 0.001). Independent experiments were performed in triplicate. Scale bar = 50 μm.

The association between CAFs and tumor cells is extremely important in understanding TME. Therefore, we co-cultured GFP-transduced human PaC cells, MIA PaCa-2-GFP (***Figure 2A***) and Capan1-GFP (***Figure 2E***), with CAFs to better identify each cell type under 3D culture condition. In ultra-low attachment plates and sphere-induction media, the size of 3D spheres increased continuously over time and reached more than 30 times the cell fold-change at day 16. The statistical significances among each time point were observed (*p < 0.05, **p < 0.01, and ***p < 0.001). To examine whether our induced tumor-CAF spheres exhibit CSC properties, we further assessed the expression of CD44 and CD133 within the co-cultured spheres, MIA PaCa-2 + CAF (***Figure 2B to 2D***) and Capan1+ CAF (***Figure 2F to 2H***). Our results showed that the formation of a niche-like environment with high CD44 and CD133 levels, which not only facilitated sphere growth but also promoted stemness characteristics.

### 3D multicellular cancer spheroids exhibit enhanced tumorigenicity and tumor invasion compared to that of 2D-spheroids

**Figure 3:**
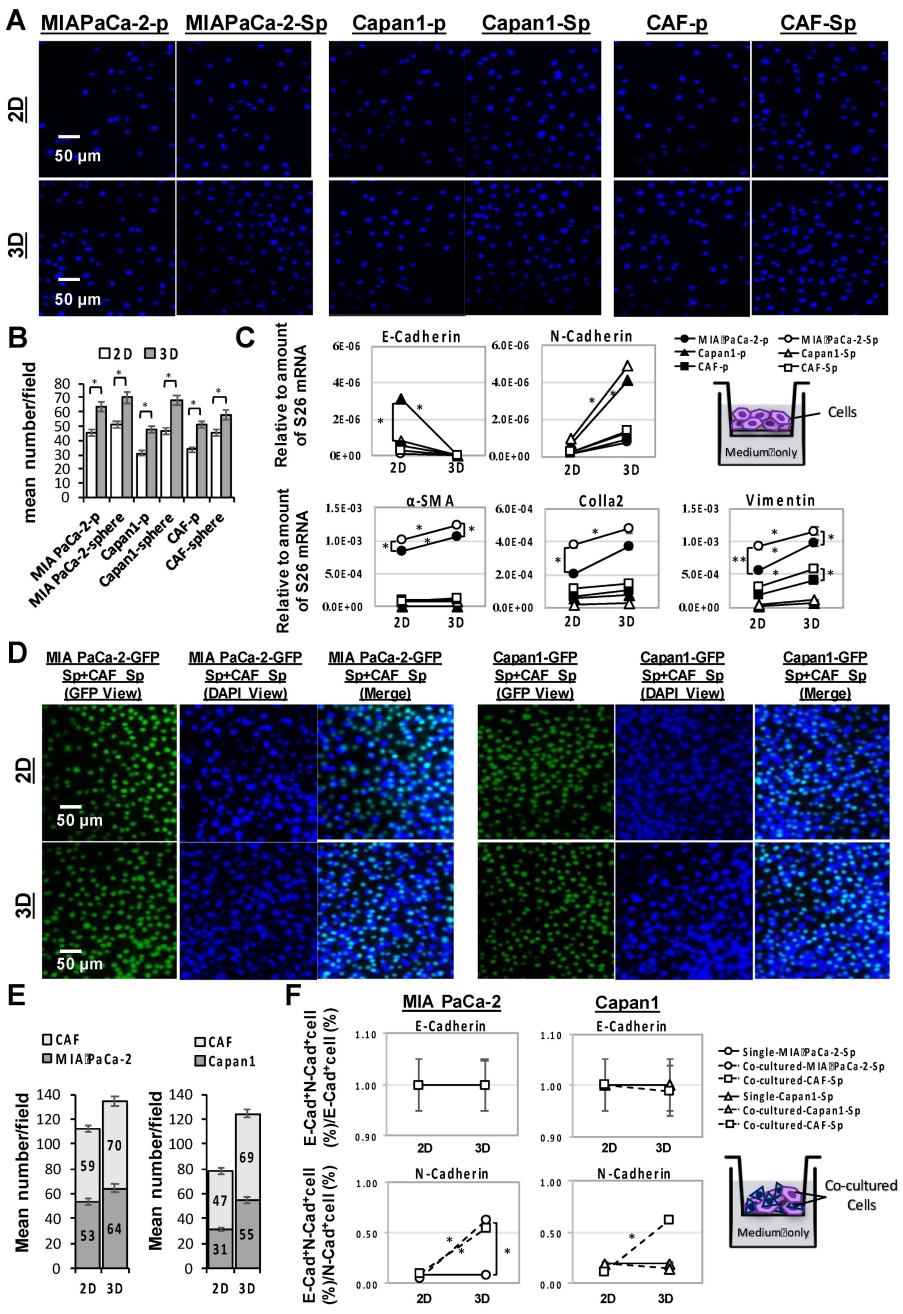
** Enhancement of cellular invasion and expression profile alterations in PaC and CAF cultured alone or in combination in 3D- compared to 2D-culture system.** Human PaC cells, MIA PaCa-2 and capan1, and CAF cells in parental (p)- and sphere-type obtained from 2D or 3D culture were used for invasion assays. (A and B) A significantly increased invasion ability of cells from 3D culture compared to those from 2D culture conditions by DAPI staining (mean number/field; *p < 0.05). Scale bar = 50 μm. **(C)** A Higher expression of α-smooth muscle actin (α-SMA), collagen 1A2 (COL1A2), vimentin, N-Cadherin and a lower expression of E-Cadherin were associated with a 3D culture condition. MIA PaCa-2 cell spheroid culture demonstrated a higher expression of higher expression of α-SMA, COL1A2 and vimentin. The invasive capabilities were also performed by GFP-transduced PaCs co-culture with CAFs in 2D and 3D conditions comparison. Similar results to individual cell lines were obtained, (D and E) mean cell numbers under a 3D condition were significantly increased than those under a 2D condition. **(F)** Both E- and Ncadherin levels remained unchanged in single tumor sphere culture under a 2D or a 3D culture condition. A significant increment of N-cadherin expression and a relatively stable E-cadherin expression in response to a CAF-co-cultured-MIA PaCa-2 sphere and to both MIA PaCa-2 and Capan1 co-cultured-CAF spheres under 3D multicellular culture conditions compared to 2D culture conditions. Data are means ± SD of triplicate independent experiments (*p < 0.05; **p < 0.01). Scale bar = 50 μm.

A comparison of cell invasion capacity between cultivation in a 3D multicellular and 2D monolayer of MIA PaCa-2, capan1, and CAF cells under individual (***Figure 3A***) or co-cultured (***Figure 3D***) conditions regarding their mean invasive cell numbers (***Figure 3B and E***) and the change of (m)RNA expression profile including alpha-smooth muscle actin (α-SMA), collagen-1-alpha 2 (COL1A2), vimentin, E-cadherin, and N-cadherin which carried out by q-PCR assays (***Figure 3C and F***). Quantifying invasive cells by DAPI-staining in each cell condition using immunofluorescence microscopy analysis indicated that the mean invasive cell numbers under 3D conditions were significantly higher than those under 2D conditions (*p < 0.05; ***Figure 3A and 3B***). Furthermore, significant COL1A2 (m)RNA expression was paralleled by an increment of α-SMA, Vimentin, and N-Cadherin (m)RNA under a 3D culture condition, whereas E- cadherin was decreased. Regarding spheroid structure, it was accompanied by high COL1A2, α-SMA, and Vimentin, especially in the MIA PaCa-2 cell line (*p < 0.05;*** Figure 3C***).

Examination of the cell surface E-cadherin and N-cadherin expressions of PaC and CAF parental- and sphere-type cells from 2D or 3D culture conditions were determined by flow cytometry. E-cadherin+N-cadherin+ cells (%) to E-cadherin+ cells (%) and to N-cadherin+ cells (%) ratios represented the altered compositions of the E- and the N-cadherin, respectively. The proportion of N-cadherin expressions trended upward significantly in all three PaC and CAF 3D cultured sphere-type cells, compared to 3D sophisticated parental (p)-type cells and to 2D cultured sphere-type cells, while it remained stationary for E-cadherin expressions regardless of the cell type or the culture setting (***Supplementary Figure 4***).

The invasion capabilities were further analyzed by GFP-transduced PaCs coculture with CAFs in 2D and 3D conditions (***Figure 3D to 3F***). GFP tagged the protein expression of invaded cells in the lower membrane, while the nuclei of invaded cells were counterstained by DAPI (***Figure 3D***). Cell surface E- and N-cadherin expressions were examined for further confirmation by flow cytometry analysis. The results showed a significant increment of mean invasive cell numbers in response to 3D multicellular culture compared to 2D monolayer culture (***Figure 3E***). Regardless of a 2D or 3D culture condition, a single PaC sphere culture demonstrated neither E- or N-cadherin level change. However, a significant increment of N-cadherin expression and a relatively stable E-cadherin expression in response to a CAF-co-cultured-MIA PaCa-2 sphere and both MIA PaCa-2 and Capan1 co-cultured-CAF spheres under 3D multicellular culture conditions compared to 2D culture conditions was observed (*p < 0.05; ***Figure 3F***). Increased expression of epithelial-to-mesenchymal transition (EMT)-related proteins, such as N-Cadherin, in PaC spheroids is dependent on the associated CAF co-culture under 3D conditions. Taking a step further, CAFs play the role of tumor-forming niche cells to interact with cancer cells and provide spatial structure for cellular support to form a functional 3D tumor sphere in our co-culture system.

### Chemokine and chemokine receptor involved in T-cell migration toward 3D spheroid culture

**Figure 4:**
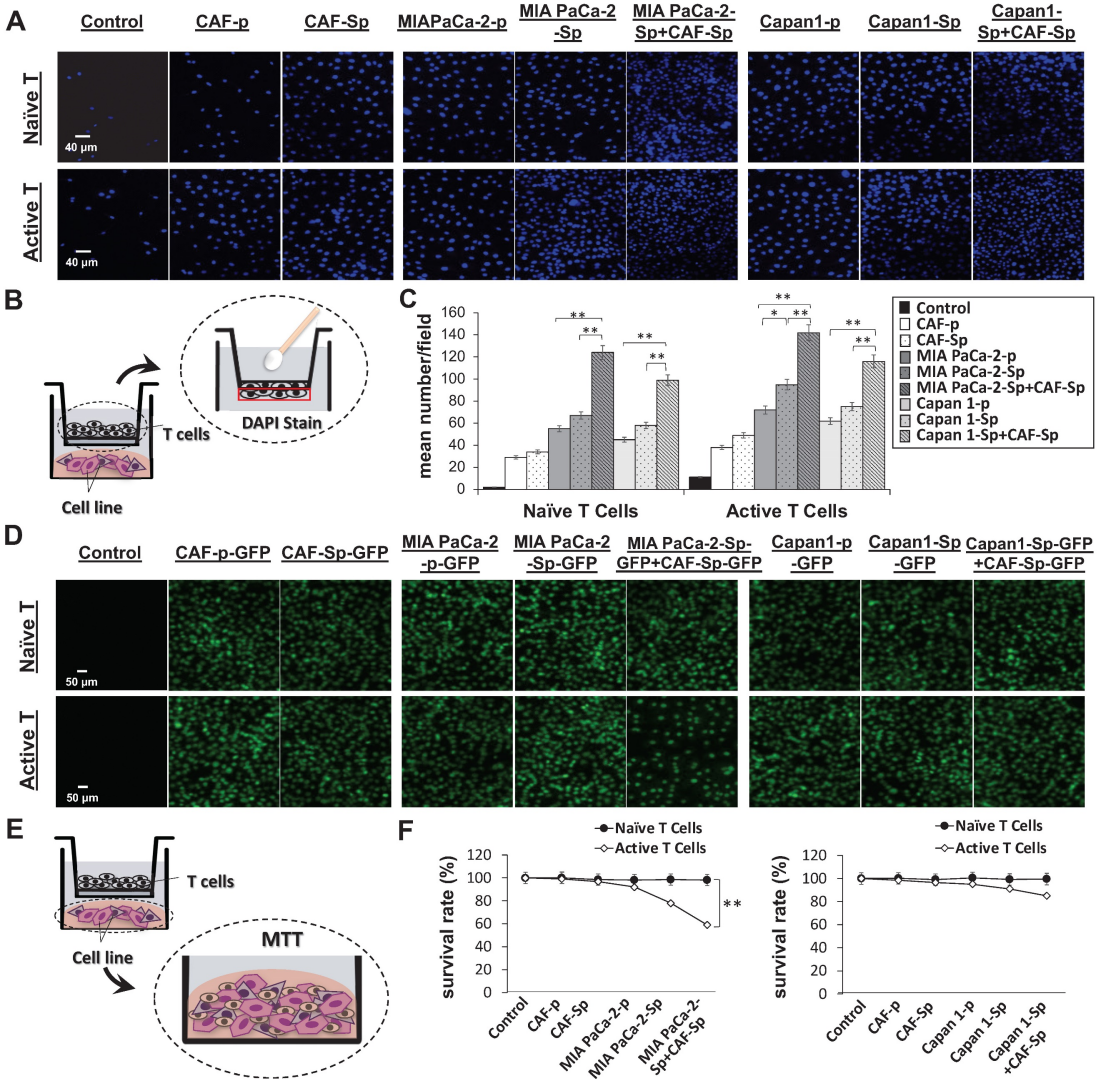
** A significantly increased active T-cell recruitment and cytotoxicity toward MIA PaCa-2 cells co-cultured with CAF in a 3D culture condition.** T-cell chemotaxis was assayed using 24-well transwell chambers with Matrigel-coating membranes. (A and B) The significant migration of both naïve T cells and active T cells into the lower chamber containing different indicated cell types in a 3D culture conditions by DAPI staining. The scale bar shown is 40 μm. CAF-integrated pancreatic cancer cell spheroids (MIAPaCa-2-sphere+CAF or Capan1-sphere+CAF) were more significant than in other groups. (C and D) The significant cytotoxic activity of migrated active T cells toward MIAPaCa-2+CAF co-culturing compared to the other cell conditions by using MTT assays, indicating that co-culture of MIAPaCa-2 with CAF created a suitable condition to enhance immune cell infiltration toward cancer cells. Data presented are the mean ± SD of triplicate independent experiments (*p < 0.05; **p < 0.01; ***p < 0.001). Scale bar = 50 μm.

**Figure 5:**
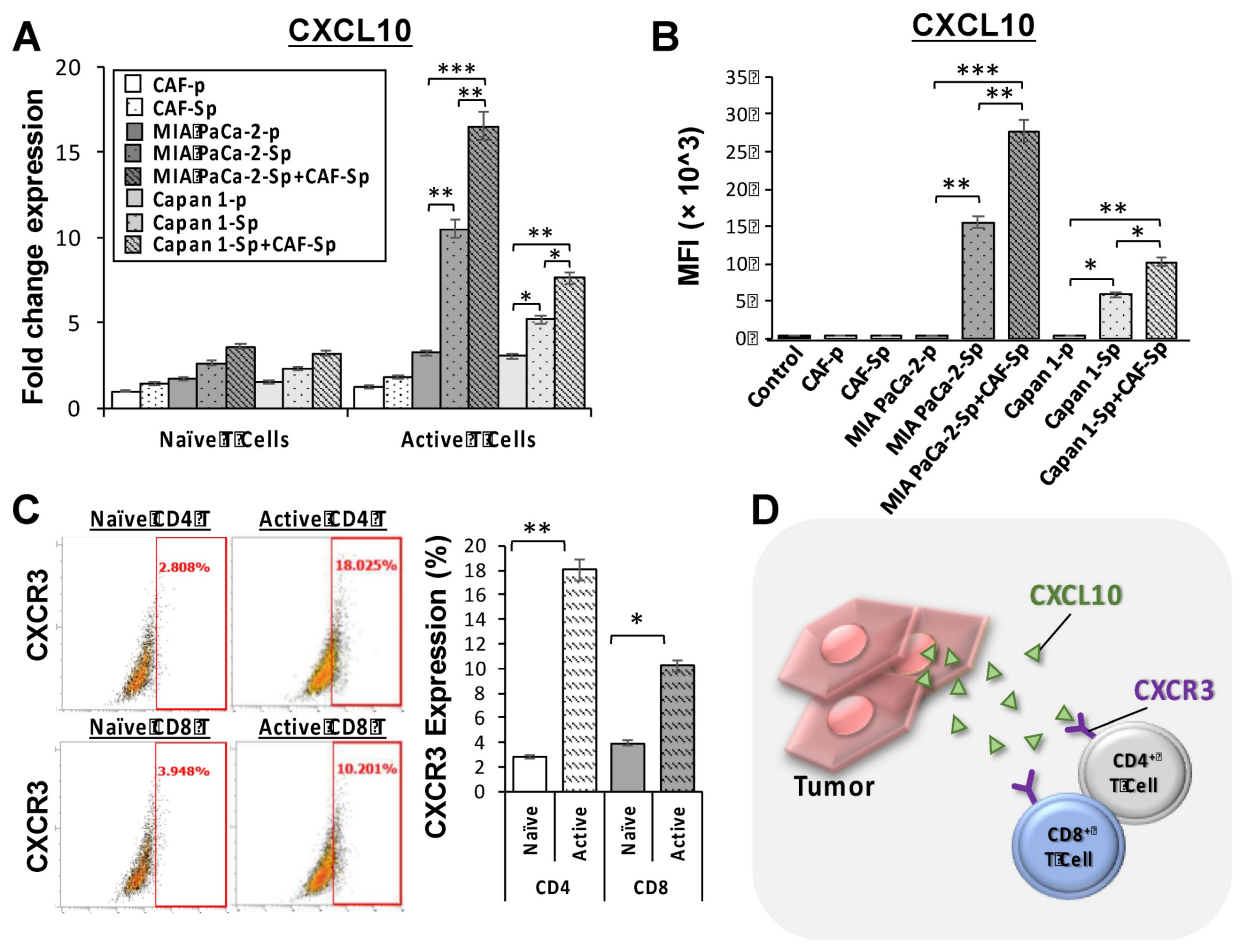
** MIA PaCa-2 cells co-culture with CAF cells facilitated T-cell recruitment through CXCR3/CXCL10 axis. (A)** CXCL10 ligand expression was significantly increased in PaC spheroids, MIA PaCa-2-sphere and Capan1-sphere, alone and co-cultured with CAF cells, as well as **(B)** in their cultured medium by using qPCR assays (mRNA fold change) and flow cytometry analysis (MFI), respectively, in 3D culture conditions. Furthermore, **(C)** cell surface receptor CXCR3 levels were significantly increased in both active CD4+T and CD8+ T cells compared to their naïve populations using flow cytometry analysis, illustrating **(D)** T cell recruitment via the CXCR3/CXCL10 signaling pathway. Data are means ± SD of triplicate independent experiments (*p < 0.05; **p < 0.01; ***p < 0.001).

We studied naïve and active T-cell recruit ability using a transwell assay to differentiate between distinct culture conditions and T-lymphocyte chemotaxis. Migrated T-cells toward cultured cancer cells were stained with DAPI, which allowed us to perform quantitative analysis of migrated T-cell numbers under defined environments. Compared to other culture conditions, the chemotactic responses became apparent from CAF-integrated PaC cell spheroids in MIA PaCa-2-sphere+CAF and Capan1-sphere+CAF cocultured settings, compared to other culture conditions (***Figure 4A and 4B***). Naïve T cells as well as activated T cells were capable of being recruited across the transwell membrane. A cell viability assay was carried out to clarify whether T-cells migrated from top chambers (maintained their property of cytotoxicity). Activated T-cells induced significant apoptosis of MIA PaCa-2 cells co-cultured with CAFs under a 3D culture condition (**p < 0.01; ***Figure 4C and 4D***). It is believed that tumor cells evolved to express some chemokine receptors, such as CXCR3, and secrete its ligand CXCL10. Moreover, CXCL10 can recruit CXCR3+ tumor-infiltrating CD4+ T cells and CD8+ T cells that are likely to enhance anti-cancer response.^23^ To investigate how the CXCR3/CXCL10 axis involved T cells recruitment in the TME, we first confirmed CXCR3 expression in a 4-day induction sphere by flow cytometric analysis. The expression of CXCR3 in spheres has augmented levels compared to a non-spheroid structure (***Supplementary Figure 5***). We thus demonstrated a possible interaction of CXCR3 expressed by activated T cells and CXCL10 secreted by tumor cells. We found that CXCL10 expression and secretion were significantly increased in CAF-integrated PaC spheroids (MIA PaCa-2- sphere+CAFs or Capan1-sphere+CAF) than in other cell groups stimulated with activated T cells. The expression level of CXCL10 was significantly higher in the CAF-integrated PaC spheroids compared to other cultured cells (***Figure 5A***). Moreover, medium CXCL10 levels were normalized by calculating the quantification ratio mean fluorescence intensity (MFI) between medium from various cultured cells (***Figure 5B***). While absent on naïve T cells, higher expression of CXCR3 was observed on either active CD4^+^ or CD8^+^ T cells, as demonstrated by flow cytometry. The positive cells in the right portion are the percentages of totally gated cells (***Figure 5C***). The illustration shows the effect of CXCL10 on chemotaxis and T-cell recruitment. Highly expressed CXCR3 chemokine receptors on active T cells are activated by interferon-inducible ligands CXCL10. CXCL10 ligands prompt immune cell migration and activated T cells possibly induce upregulation of chemokine, including CXCL10 (CXCR3-mediated). The results suggest that this axis could form a positive feedback loop that amplifies the initiating stimuli between CXCL10 and its receptor CXCR3 (***Figure 5D***).

Regarding the external controls rather than PaC cells, as illustrated in ***Supplementary Figure 6***, significant increases of CXCL10 mRNA fold changes on qPCR assays and CXCL-10 (IP-10) concentrations on ELISA assays were seen in the SKOV3-spheres alone and co-cultured with CAF cells, especially when being stimulated with activated T cells (with higher CXCR3 expression as shown in Figure 5C). It seems that the SKOV3 sphere and its co-culture with CAFs are capable of facilitating T-cell recruitment through the CXCR3/CXCL10 axis. Nevertheless, no similar situation was observed in the WS1 cells.

### Acquired chemoresistance in 3D PaC spheres

**Figure 6:**
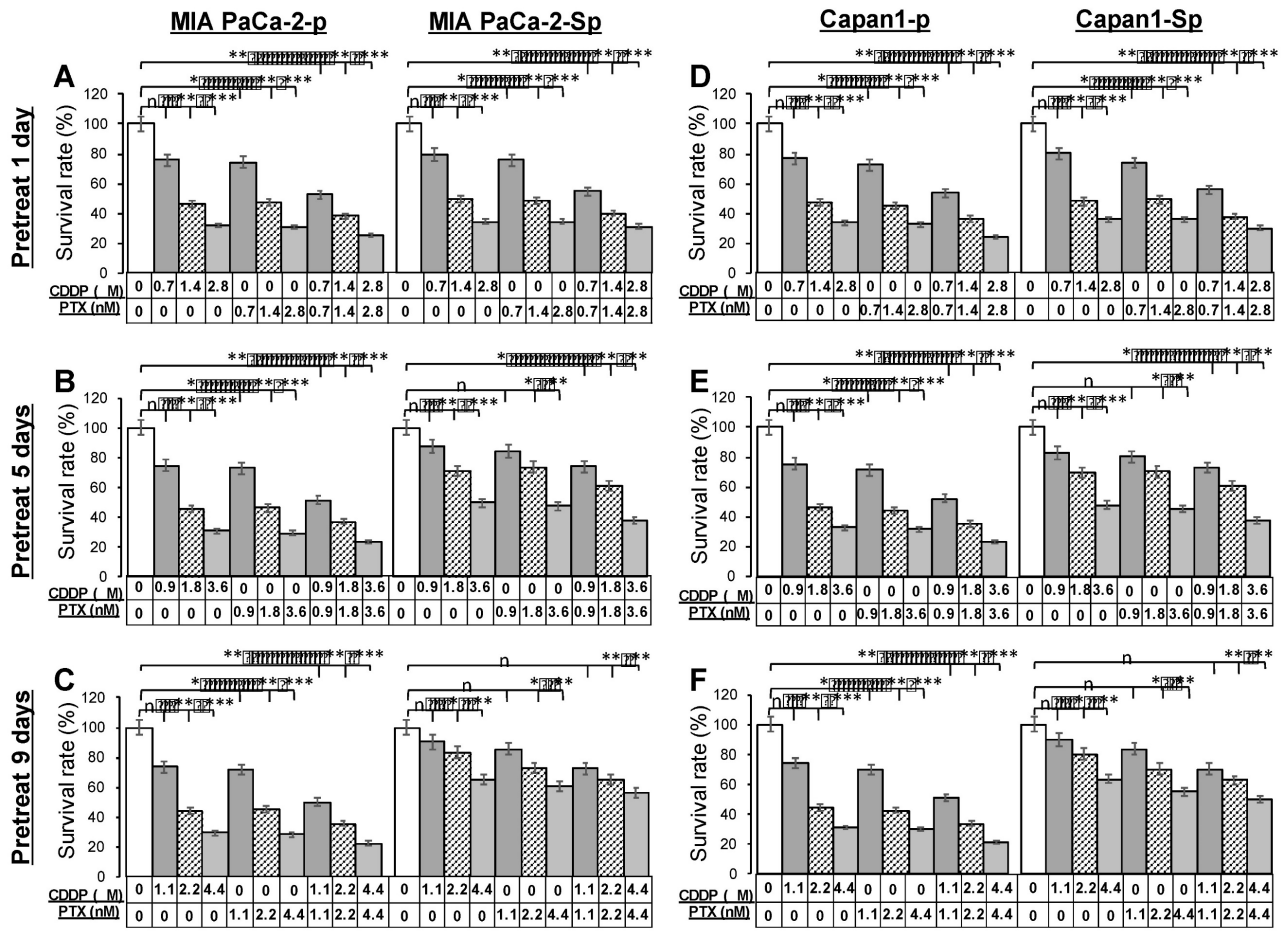
** Pretreatment with paclitaxel (PTX) combined with cisplatin (CDDP) synergistically enhanced the chemoresistant capacities of PaC-spheres in 3D culture conditions.** MIA PaCa-2 parental cells (MIA PaCa-2-p) and Capan1 parental cells (Capan1-p) were pretreated with 1 nM PTX and 1 μM CDDP in combination for 1, 5, and 9 days, and then cells were collected for a 4-day sphere-forming in 3D culture conditions. (A to C) MIA PaCa-2-p and -sphere as well as (D to F) Capan1-p and -sphere populations were sorted for cell viability assay comparisons among untreated, CDDP titration-treated, PTX titration-treated, and CDDP/PTX titration-treated after 48 h of drug treatment. Presented data were acquired from three independent experiments (n, no significant difference, *p < 0.05, **p < 0.01, and ***p < 0.001).

The PTX and CDDP are often combined for their anti-cancer ability with tolerable clinical safety.^24^ Before analysis of cytotoxicity profiles toward anti-cancer drugs, MIA PaCa-2 parental cells (MIA PaCa-2-p) and Capan1 parental cells (Capan1-p) were pretreated with 1 μM CDDP and 1 nM PTX in combination for 1, 5, and 9 days. To evaluate the chemoresistant ability, parental cells (***Figure 6***, left portions) and derived CSC-like spheres under 3D culture conditions for four days (***Figure 6***, right portions) were exposed to a range of drug concentrations of CDDP, PTX, and CDDP/PTX for 48 hours. The remaining viable cells from different drug-titration groups were assessed by an endpoint MTT assay and compared. Dimethyl sulfoxide (DMSO) was used as an untreated control. Parental cells remained sensitive, significantly reducing viable cells after exposure to most therapeutic titration, regardless of prolonged drug pretreated days. On the contrary, dose-dependent chemoresistance in 3D spheres developed with the addition of protracted pretreated days. A decrease in drug sensitivity was observed at a relatively low dose of drug 0.7 μM CDDP (drug pretreated for one day), 0.9 μM CDDP and 0.9 nM PTX (drug pretreated for five days), 1.1 μM CDDP, 1.1 nMPTX, and 1.1 μM CDDP and 1.1 nM PTX in combination (drug pretreated for nine days) compared to the untreated group. The results demonstrated that 3D sphere cells exhibited more excellent capability of anti-cancer drug resistance. Furthermore, the two PaC cell lines, MIA PaCa-2 and Capan1; were cultured in 2D and 3D conditions. The cell viability data revealed similar half maximal inhibitory concentration (IC50) patterns for the two cell lines tested with CDDP and PTX in different concentration ranges of 1.0-2.2 μM and 1.0-2.2 nM, respectively. The IC50 of CDDP and PTX inMIA PaCa-2 and Capan1 under 2D culture conditions derived from days 1, 5, and 9 were 1.0 μM and 1.0 nM, 1.4 μM and 1.4 nM, 1.8 μM and 1.8 nM, respectively. The IC50 of CDDP and PTX in MIA PaCa-2 under 3D culture conditions derived from days 1, 5, and 9 were 1.4 μM and 1.4 nM, 1.8 μM and 1.8 nM, 2.2 μM and 2.2 nM, respectively (***Supplementary Figure 7A and 7B***). The measured IC50 values of drugs were positively correlated with longer culture times and cells cultured under a 3D condition.

### Cytotoxic efficacy of protease-activated receptor (PAR)1CAR-T cells toward chemoresistant CAF-integrated PaC spheres

**Figure 7:**
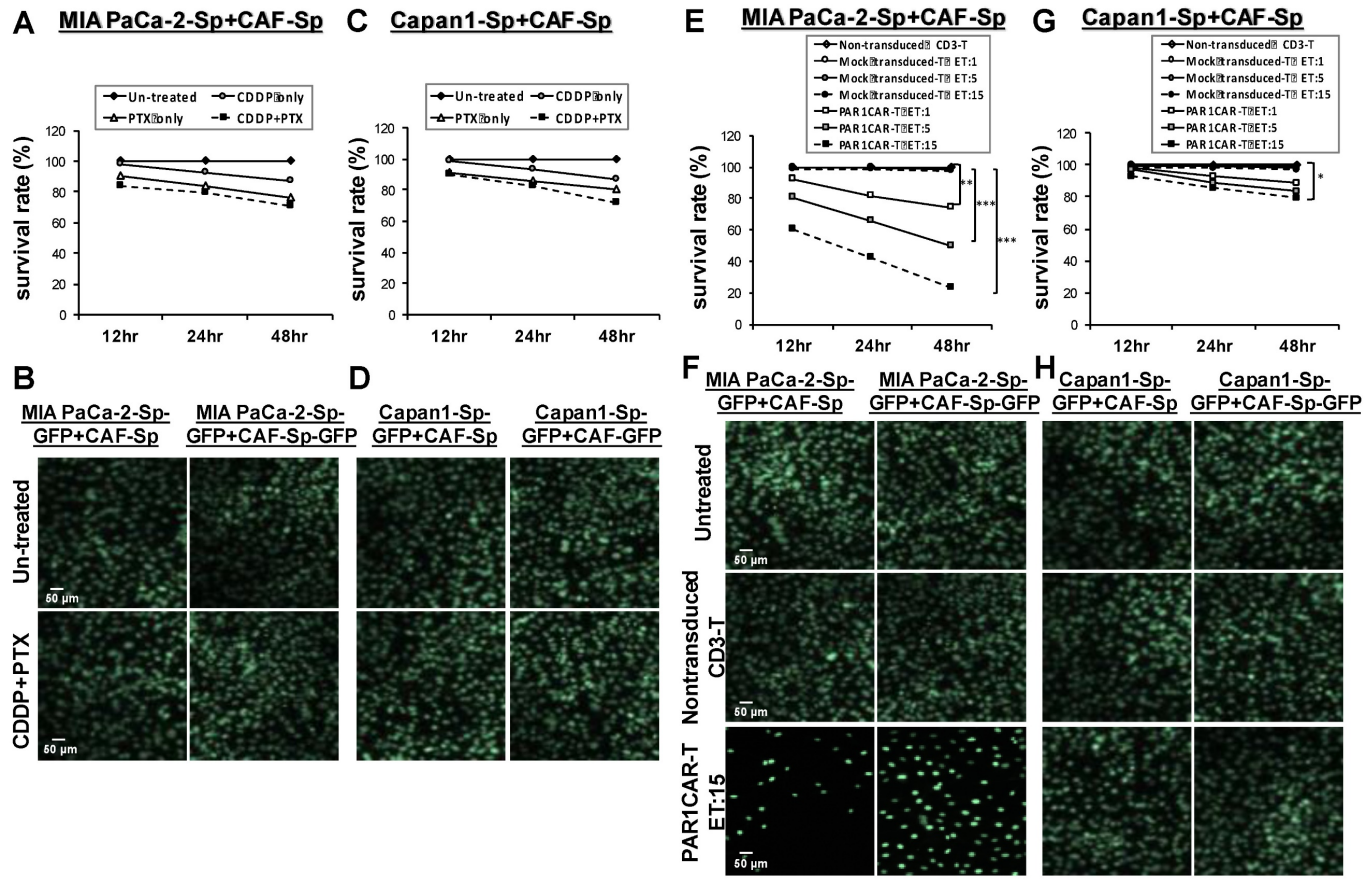
** Significant cytotoxicity activities of PAR1CAR-T cells toward chemoresistant PaCs co-cultured with CAFs in 3D culture conditions.** Development of chemoresistance in (A and B) MIA PaCa-2 cells as well as (C and D) Capan1 cells co-cultured with CAF cells in 3D culture condition. Cell viability of cancer spheroids in response to CDDP, PTX, or their combination did not differ compared to untreated controls following 12 h, 24 h, and 48 h treatment by using (A and C) MTT assays and (B and D) GFP fluorescence images. However, the same 12 h, 24 h, and 48 h cytotoxicity activities were performed using MTT assays with at least three replicates with increasing effector (PAR1-CAR-T cells) concentrations to target E:T ratios of 1, 5, and 15 against 3D cancer spheroids **(E)** MIA PaCa-2+CAF and **(G)** Capan1+CAF. Cytotoxic activities were compared with non-transduced CD3 T cells, and mock-transduced CD3 T cells served as the controls of chimeric modified T cells (*p < 0.05, **p < 0.01, and ***p < 0.001). Significant cytotoxicity results of PAR1CAR-T cells toward 3D cancer spheroids **(F)** MIA PaCa-2+CAF and **(H)** Capan1+CAF were also observed using GFP fluorescence imaging. Data presented are the mean ± SD of triplicate independent experiments. Scale bar = 50 μm.

In ***Supplementary Figure 8***, the endogenous PAR levels in viable tumor cells were evaluated by flow cytometry analysis. The proportion of cells with PAR1 expression in MIA PaCa-2 cells was considerably higher than the ones in Capan1 cells (97.024% versus 3.589%); PI was used to exclude non-viable cells in flow cytometry. MIA PaCa-2 cells (***Figures 7A and 7B***), as well as Capan1 cells (***Figures 7C and 7D***) were co-cultured with CAF cells in 3D culture condition. MTT assays assessed cell viability in response to CDDP, PTX, or their combination at 12 h, 24 h, and 48 h. Cell survival rates did not differ compared to the untreated control group in both cell lines (***Figures 7A and 7C***). GFP immunostaining of CAF-integrated PaC spheroids in response to CDDP and PTX combination revealed static compared to the untreated control group (***Figures 7C and 7D***). We demonstrated killing dynamics of non-transduced T cells, Mock-T cells, and PAR1CAR-Tcells at different effector to target (E:T) ratios (1:1, 5:1, and 15:1) against CAF-integrated PaC spheroids. The time scale was set up at 12 h intervals for display. Non-transduced T cells served as control reference, and Mock CAR-T cells' survival lines (curves) overlapped. PAR1-CAR-T cells exhibited enhanced cytotoxic ability at any E:T ratios in MIA PaCa-2-sphere+CAF cultured cells. Immunofluorescence analysis supported scattering cells observed after exposure by PAR1CAR-T cells with an E:T ratio of 15 (***Figures 7E and 7F***). PAR1CAR-T cells showed significant inhibition of Capan1-sphere+CAF cultured cells only at a high E:T ratio of 15 (***Figures 7G and 7H***), (*p < 0.05, **p < 0.01, and ***p < 0.001). The viability of chemoresistant tumor spheres was significantly reduced by nearly 80% and 20% upon PAR1CAR-T cells at a high E:T ratio in MIA PaCa-2- sphere+CAF and Capan1-sphere+CAF cultured cells, respectively. The results implied that the response of 3D spheres toward PAR1CAR-T cells therapy may be irrelevant to chemoresistance; and also indicated that PAR1CAR-T cells may be the subsequent possible treatment when chemotherapy fails.

## Discussion

To recreate an *in vitro* TME as realistic as possible has been a significant obstacle for cancer research. We established a model of a CAF-integrated PaC sphere under a 3D culture condition, which is appropriately stemness-featured and capable of reflecting a simulated TME. A 3D culture model surpasses a 2D model for having sensible and practical aspects, including cell morphology, proliferation, differentiation, and multi-functional metabolism, and is more effective in developing medications, seeking biochemical signal transduction, and evaluating therapeutic response 25-27.

The addition of CAFs in 3D model construction incorporates well with human PaC cell line cells. The architects of stroma remodeling induce the tumor cells displays tumor-like morphology, and immunological characteristics, induces the infiltration of tumor-associated lymphocytes, and generates a complicated TME 28, 29. These all play critical roles in leading to treatment insensitivity and aberrant cell apoptosis. Therefore, it is essential to reconstruct an imitating model properly 30, 31. Essentially, CAF creates a network of connections between immune-infiltrating cells and the tumor that promotes metastasis, chemoresistance, and tumor growth 28, 32, 33. Our 3D cultured PaC spheres showed stronger chemoresistance compared to monolayer cells in 2D conditions, which provides a simulated platform for subsequent therapeutic sensitivity analysis. A recent study regarding a CAF-integrated PaC organoid model supports that deposited collagen I in the extracellular matrix counteracts the chemotherapeutic effect by blocking drug transport.^34^ It is cost-effective and time-saving to use PaC cell lines, serving tumorigenic exploration and therapeutic evaluation *in vitro*. Yet, there is still a real gap when considering clinical customization. It has been a breakthrough to overcome the obstacle to exemplifying the CAF-regulated TME. As shown in our study, spheroid formative and CAF co-culture processes were associated with tumorigenesis, cell migration and invasive ability, thus also working in concert with the causality between EMT-related marker expressions and enhanced tumor behavior in PaC 35. The reduction of E-cadherin expression 36, the increment of N-cadherin, α-SMA 37, vimentin, and COL1A2 38 signals are components of the EMT process. In the current study, an atypical EMT presentation of stationary E-cadherin expression rather than a typical decrement might result from being countervailed by the cadherin-mediated structural transformation. A spheroid structure promotes intercellular adhesion, and elevated E-cadherin might rely on increased cellular connectivity, which assists in forming tight polarized cell layers 39. EMT has far-reaching significance in PaC research. Future efforts should be put to suppress EMT, which helps to improve both treatment efficacy and patient survival.

TME is a delicate ecosystem with multiplex functions contributed by conceptual elements, cancerous, stromal, and infiltrating immune cells, working together as parts of a mechanism 40. Adding CAF in a PaC cell model alters tumor physiology and biochemical properties, and possibly plays an important role in the development of chemoresistance, which may be a consequence of recapitulating TME 34, 41. The CXCL10/CXCR3 signaling in PaC has been reported to be correlated with poor prognosis 42, 43. The cooperation between the CXCL10 ligands and CXCR3 in recruiting effector T cells, macrophages, and natural killer cells were well addressed 44, 45. Our 3D spheroid model reproduces that the CXCL10/CXCR3 axis, a key chemokine signaling in solid tumors 46, promotes T cell migration, indicating a valid constructed TME.

PaC CAR-T cell therapy has been studied with various target antigens expressed on PaC cells, including mesothelin (MSLN) 47, 48, HER-2, CD133, CD24 49, MUC1 50, prostate stem cell antigen (PSCA) 51, natural killer (NK) p46 extracellular signaling domain 52, and fibroblast activation protein (FAP) 53. Yet, reality had not lived up to expectations and a conservative attitude toward the actual anti-tumor response should be kept current 54, 55. The presence of PAR1 promote tumor growth, and the immune evasion may be mediated by T cell exclusion- independent thrombin/PAR1 signaling 56. The endogenous PAR1 expressions were 97.024% and 3.589% in MIA PaCa-2 and Capan1 cells, respectively. A high PAR1 expression is not only an influencer of cancer proliferation, invasion, and metastasis 57, 58 but also an EMT signature in PaC 59. Our model revealed the potential efficacy of PAR1CAR-T cell therapy against PaC spheroids, possessed with chemoresistance, and *in vitro* potency assays. Therefore, studying PAR1-associated tumorigenesis and disclosing its future role in molecular target therapy is indispensable.

Nowadays, 3D models, particularly for medication development, are innovative. Before performing animal trials, they enable both negative and positive selection of medication candidates, even in high-throughput circumstances. This lowers the price of developing new drugs as well as the use of laboratory animals. New directions in CAR-T cancer research can also be pursued by utilizing three-dimensional models. Also, future work is required to replicate the whole TME including the incorporation of cancerous, stromal, and more comprehensive infiltrating immune cells. The immune environment could change dynamically and develop resistance during treatment. Therefore, updated functional information of continuous and long- term culture established based on the 3D culture technique, tumor niche alteration, should be the next step to exhibit sustained tumor biology during treatment. Nevertheless, developing and expanding patient-derived organoid lines for later uses would be the limiting element for the clinical implementation of our co-culture 3D model.

## Conclusion

Our 3D constructed PaC spheroid model is an easily-reproducible CSC-like platform with scalability in studying tumor niche interaction in different therapeutic approaches in terms of conventional chemotherapy, target therapy, immunotherapy, and cell therapy to validate preclinical effects.

## Supplementary Material

Supplementary figures.

## Figures and Tables

**Table 1:** Primers

	Primer Forward	Primer Reverse
E-cadherin	TTTCCCTCAAGGCTGCAGG	ATGCCAGGAGGCCGCTCTC
N-cadherin	TTGTCATCAGCTCGCTCTCC	TATCCGGCACATGGAGGCGG
Vimentin	TAAACCGCTAGGAGCCC	ATGGCTGCGGAGGGTGG
COL1A2	CAACCAGATTGAGACCCTTCTTA	CCCGGATACAGGTTTCG
αSMA	CTATGCCTCTGGACGCACAACT	CAGATCCAGACGCATGATGGCA
CXCL10	CCAATTTTGTCCACGTGTTG	GCTCCCCTCTGGTTCCAAGG
S26	CCGTGCCTCCAAGATGACAAAG	GTTCGGTCCTTGCGGGCTTCAC

## Abbreviations

3D: Three-dimensional
TME: Tumor microenvironment
PaC: Pancreatic cancer
CSC: Cancer stem cell
CAF: Cancer-associated fibroblast
2D: Two-dimensional
iPSC: induced pluripotent stem cell
CAR: chemoresistant chimeric antigen receptor
ATCC: AmericanType Culture Collection
DMEM: Dulbecco's modified Eagle'smedium
FBS: Fetal bovine serum
STR: short-tandem repeat
GFP: Green Fluorescent Protein
EGF: Epidermal growth factor
FGF: Fibroblast growth factor
LIF: Leukemia inhibitory factor
PE: Phycoerythrin
CD: Cluster of differentiation
APC: Allophycocyanine
CDDP: Cisplatin
PTX: Paclitaxel
DMSO: Dimethylsulfoxide
DAPI: diamidino-2-phenylindole
α-SMA: alpha-smooth muscle actin
COL1A2: collagen-1-alpha 2
EMT: Epithelial-to-mesenchymal transition
P: Parental cells
IC50: Half maximal inhibitory concentration
E:T ratio: effector to target ratio
