# Supplementary material for: Enhancement of Tumorigenicity, Spheroid Niche, and Drug Resistance of Pancreatic Cancer Cells in Three-Dimensional Culture System
Source: J Cancer. 2024 Mar 2;15(8):2292–305. doi: 10.7150/jca.87494 (PMC10937281; doi:10.7150/jca.87494)
Supplement: Supplementary file 1 — Supplementary figures. [file jcav15p2292s1.pdf]

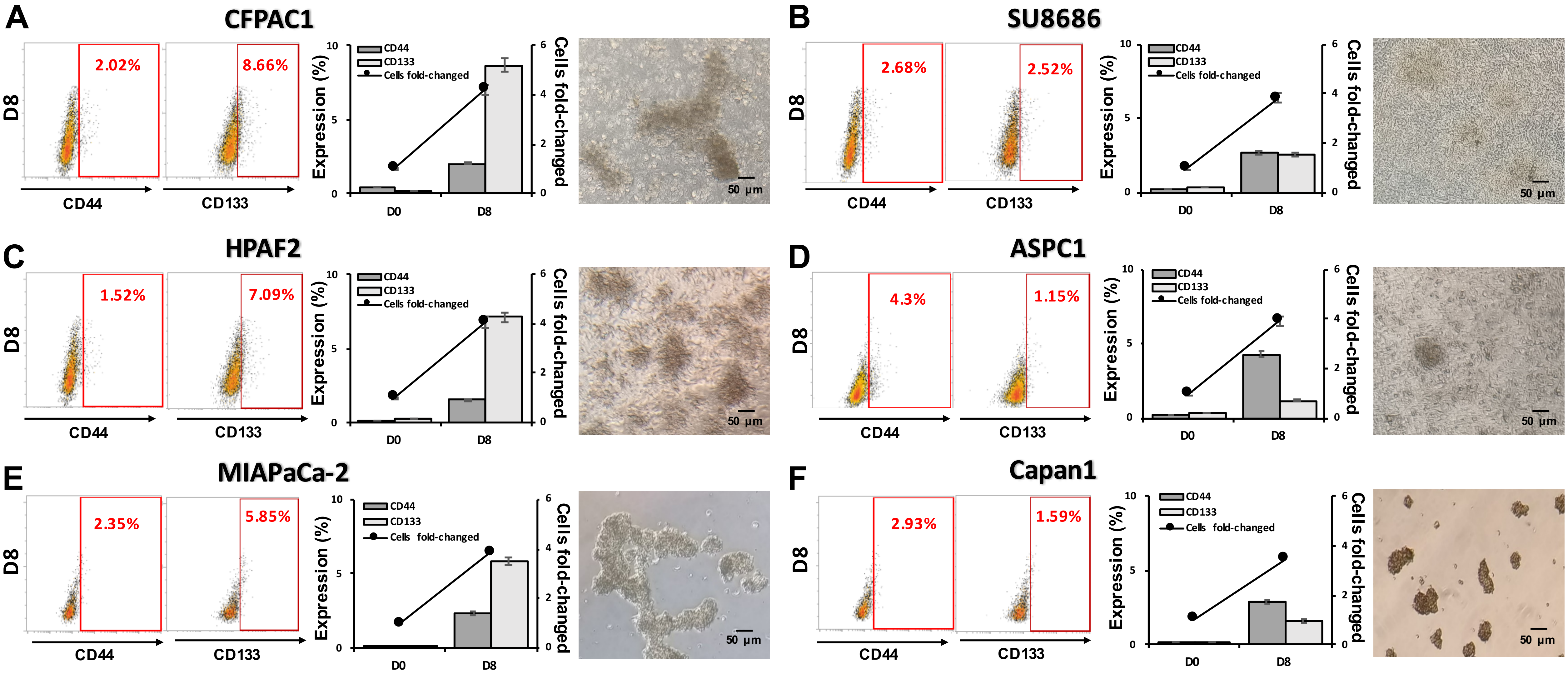

Figure S1. Morphological changes, cell-fold changes, and stemness markers expression of PaC-sphere cells derived from six PaC cell lines. PaC-sphere cells derived from human (A) CFPAC1, (B) SU8686, (C) HPAF2, (D) ASPC1, (E) MIAPaCa-2, and (F) Capan1 cells at days 0 and 8 using 2D culture system. Sphere cells were then collected at the indicated time points for cell surface CD44 and CD133 expression assessed by flow cytometric analysis. Independent experiments were performed in triplicate. Optical microscopy observed cell-fold changes correlated with CD44 and CD133 expressions and morphological changes during the sphere-induction period. Scale bar = 50 μm.

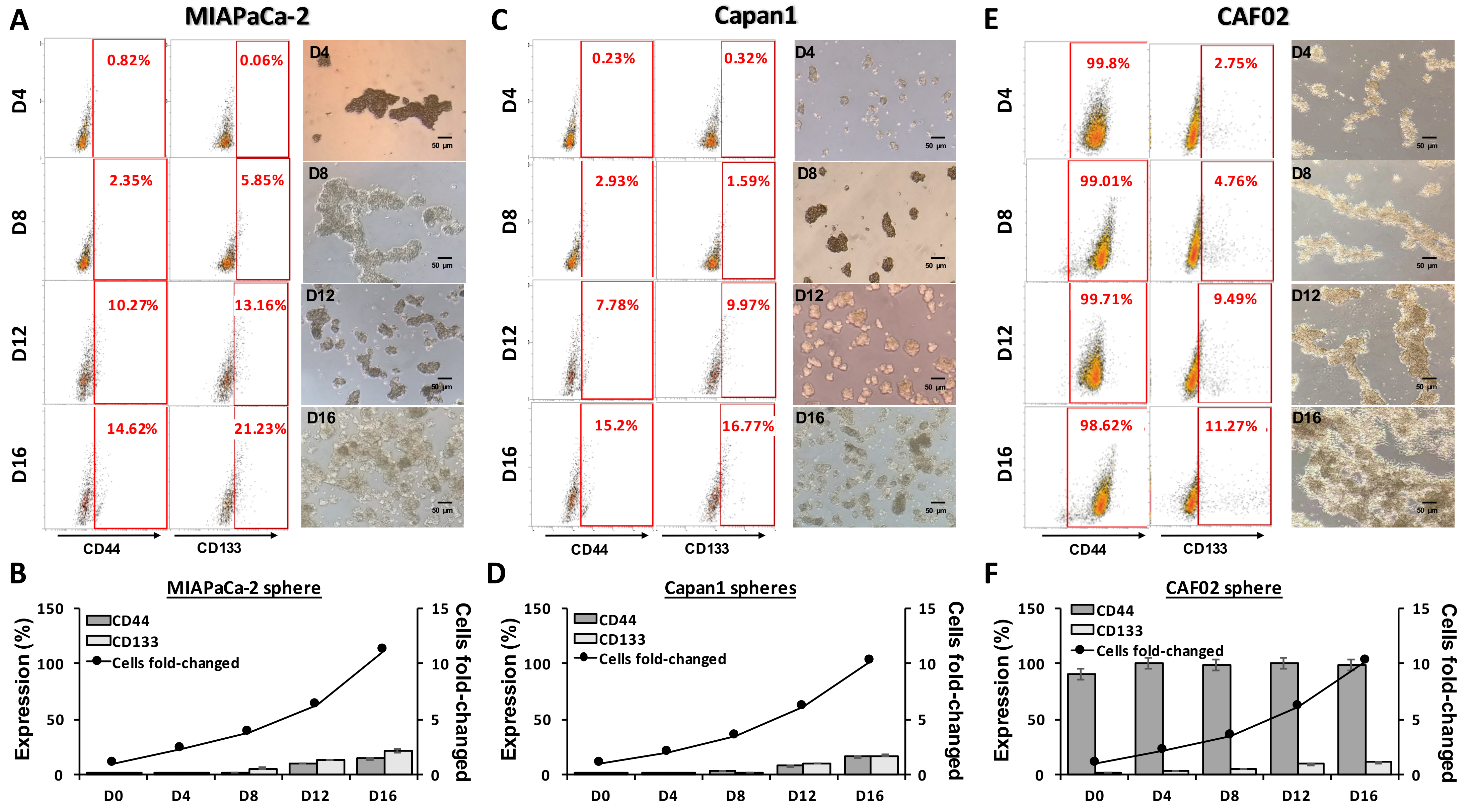

Figure S2. Morphological changes and stemness markers expression of PaC-sphere cells derived from MIAPaCa-2, Capan1, and CAF cells. PaC-sphere cells derived from human **(A and B)** MIAPaCa- 2, **(C and D)** Capan1, and **(E and F)** CAF cells at days 4, 8, 12, and 16 using 2D culture system. Sphere cells were then collected at each indicated time point for cell surface markers CD44 and CD133 expression assessed using flow cytometric analysis, cell morphological changes by optical microscopy imaging, and cell-fold change correlated with CD44 and CD133 expressions during the sphere-induction period. Independent experiments were performed in triplicate. Scale bar = 50 µm.

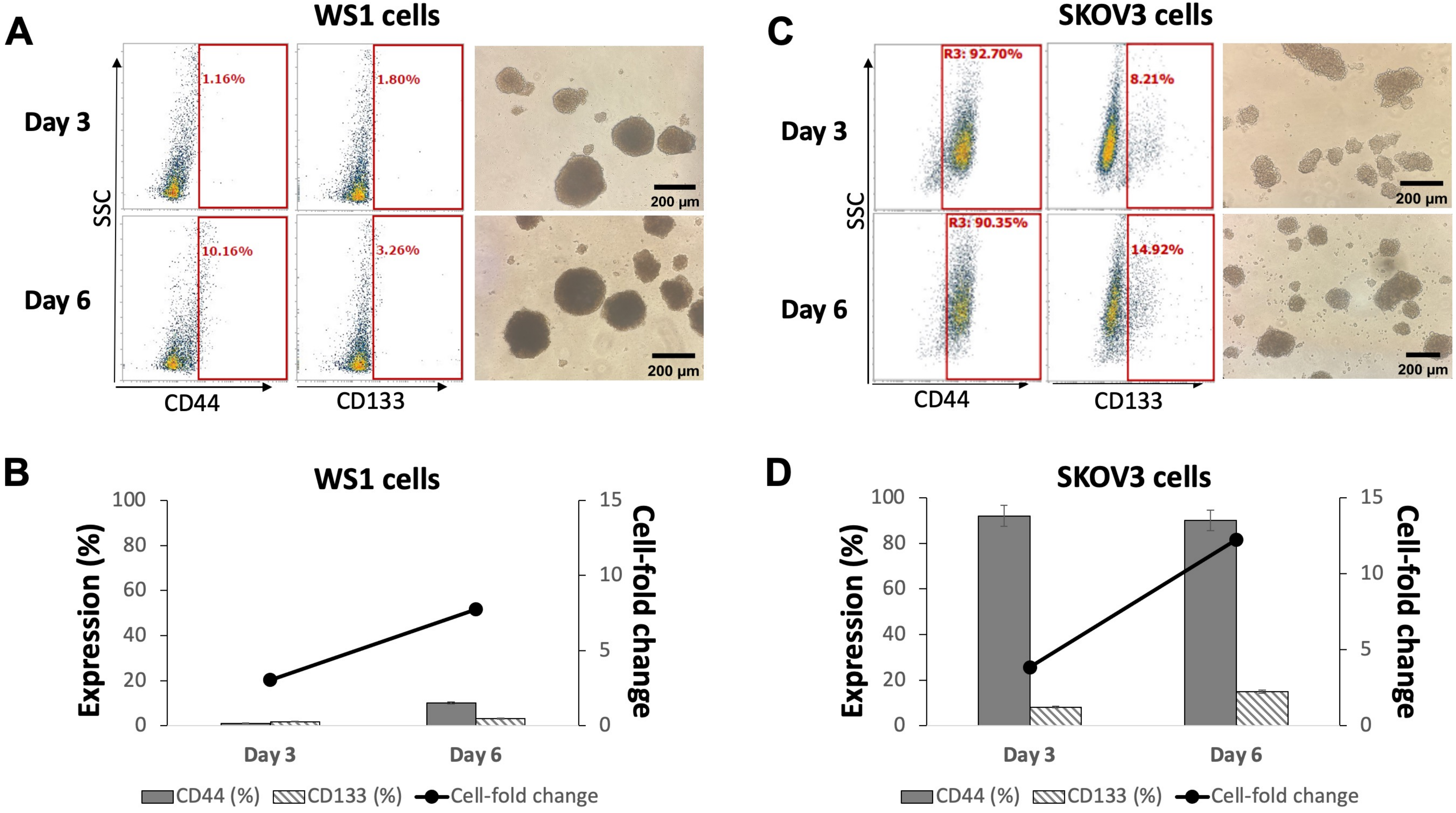

Figure S3. Significantly increased cell proliferation and essential stemness marker CD133 in SKOV3 cells using a 3D culture system. Morphological changes and stemness markers expression of the 3D-cultured normal human fibroblast **(WS1; A and B)** and ovarian cancer cell **(SKOV3; C and D)** spheres were examined. Flow cytometry characterized stem markers CD44 and CD133 of the sphere cells derived from WS1 **(A, left panel)** and SKOV3 **(C, left panel)**, respectively at day 3 and day 6 using a 3D culture system. Morphological changes of formative spheres were directly observed by optical microscopic examination during the sphere-induction period **(A and C, right panels)**. The magnitude of change in CD44 and CD133 expressions and the cell-fold change values are also estimated over 3 and 6 days **(B and D)**. Data presented are the mean ± SD of triplicate independent experiment. Scale bar = 200 µm.

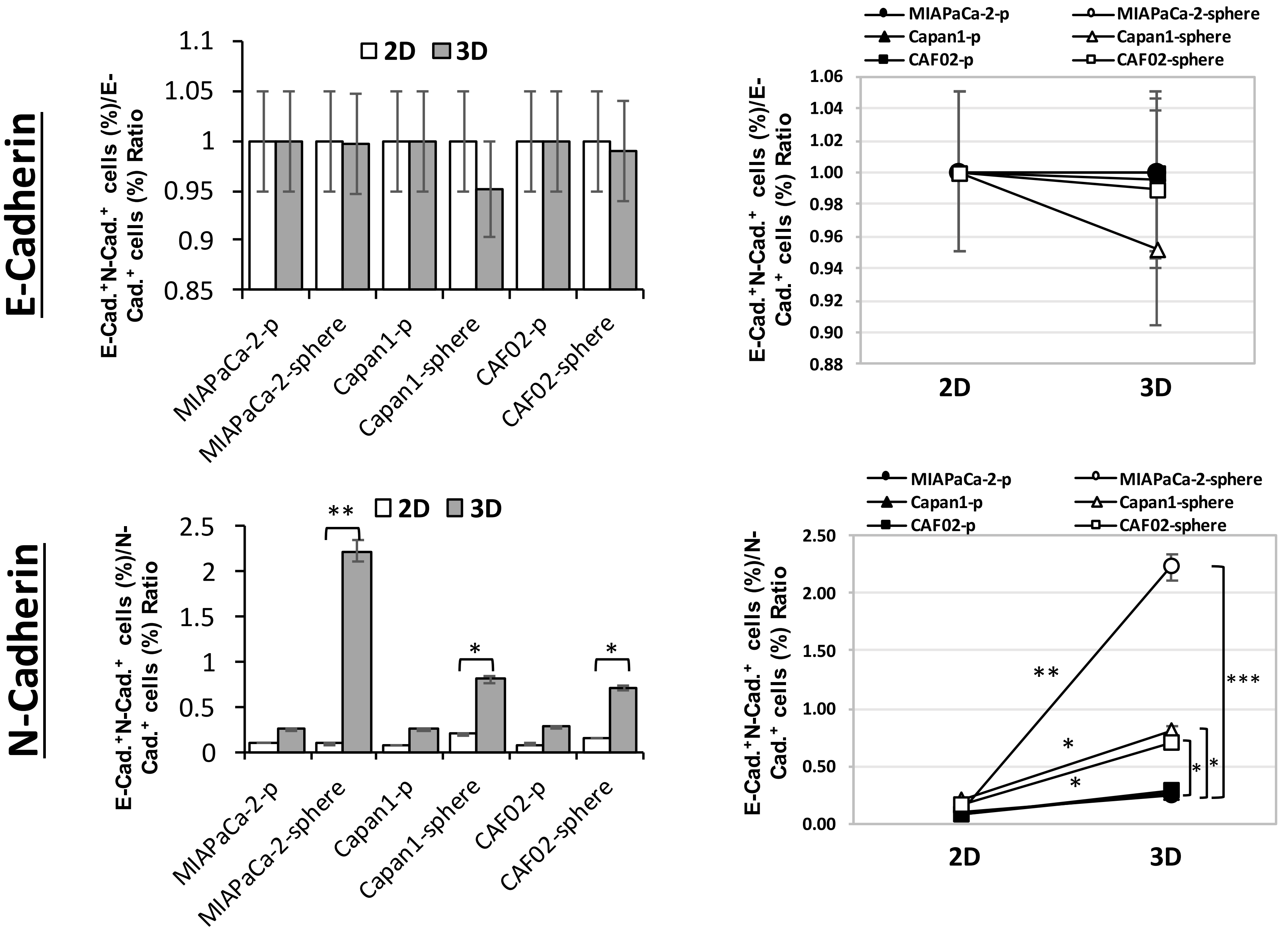

Figure S4. N-Cadherin levels were significantly increased in spheres under 3D culture system compared to 2D culture condition. Human PaC cells, MIA PaCa-2 and Capan1, and CAF cells with parental (p)-and sphere-type from 2D and 3D culture conditions were collected for cell surface E-Cadherin and N-Cadherin levels examination using flow cytometry analysis. Data represented are the ratios of E-Cadherin+Ncadherin+ cells (%) to E-Cadherin+ cells (%) and N-Cadherin+ cells (%), respectively (*$p < 0.05$; **$p < 0.01$; ***$p < 0.001$). Independent experiments were performed in triplicate.

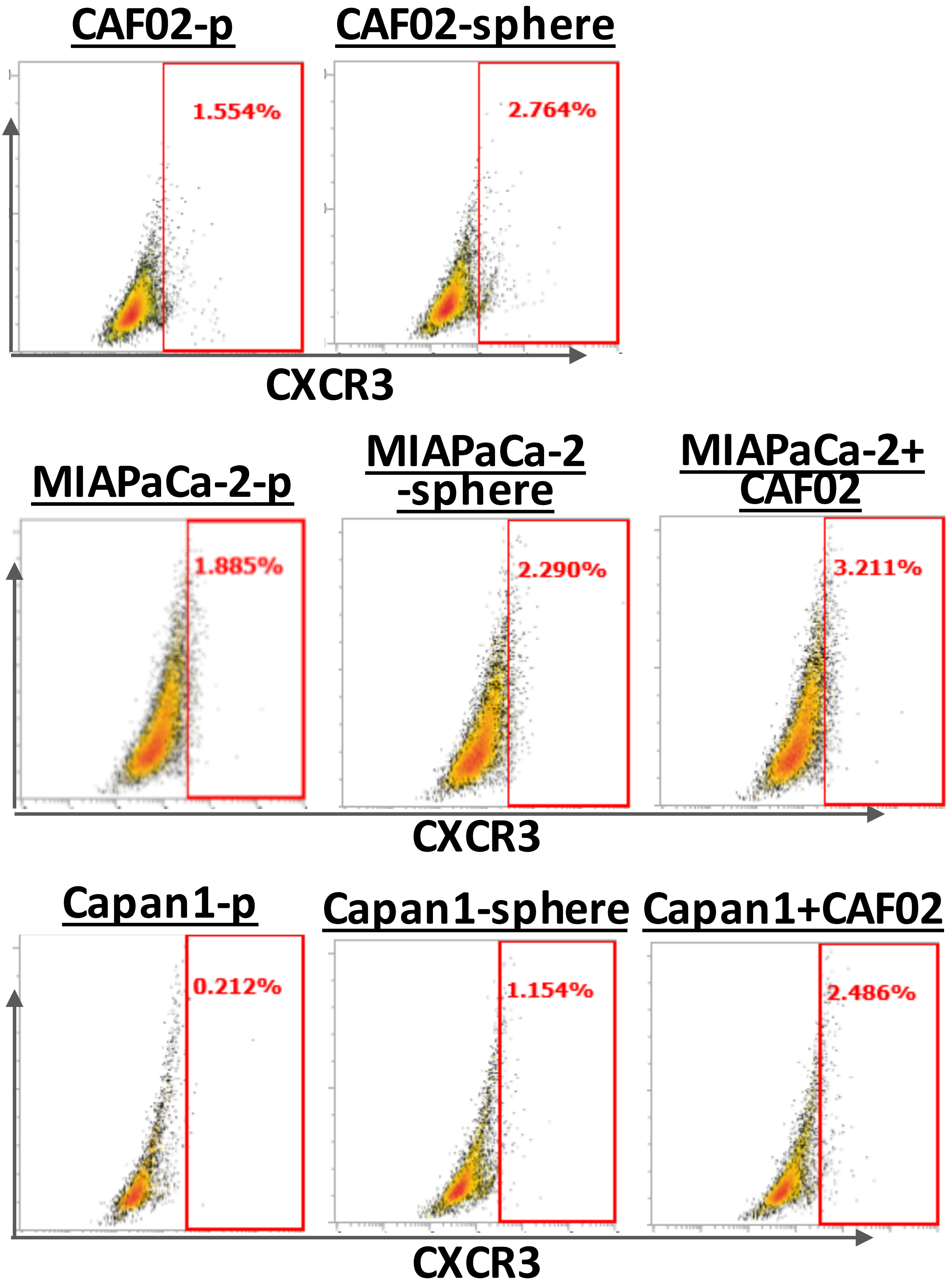

Figure S5. CXCR3 expression of PaC parental and sphere cells derived from individual or co-culture cells in 3D culture system. A 4-day induction of PaC sphere cells derived from human **(upper panel)** CAF, (**middle panel)** MIAPaCa-2, and **(lower panel)** Capan1 cells cultured either alone or in combination. Represented dot-plot results were cell surface CXCR3 expression assessed by flow cytometric analysis.

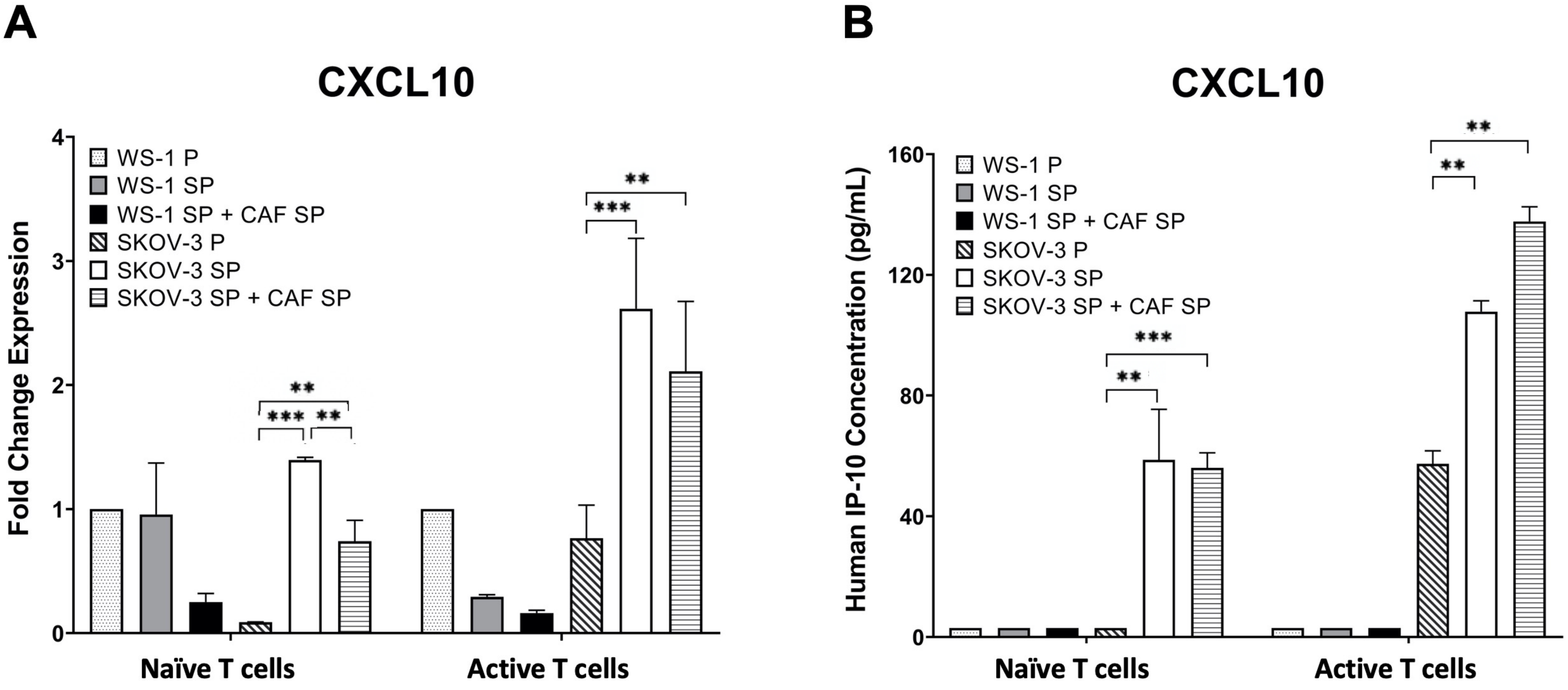

Figure S6. The WS1 and SKOV3 individual cultures in parental (p) and sphere (sp)-type and co-cultures with CAF cells. The incubation and sphere induction period were 4 days, and then check each culture for (A) CXCL-10 expression, with either activated or naïve T cell stimulation, by q-PCR assays, and for (B) CXCL-10 (IP-10) levels by ELISA assays (**$p < 0.01$, and ***$p < 0.001$). Independent experiments were performed in triplicate.

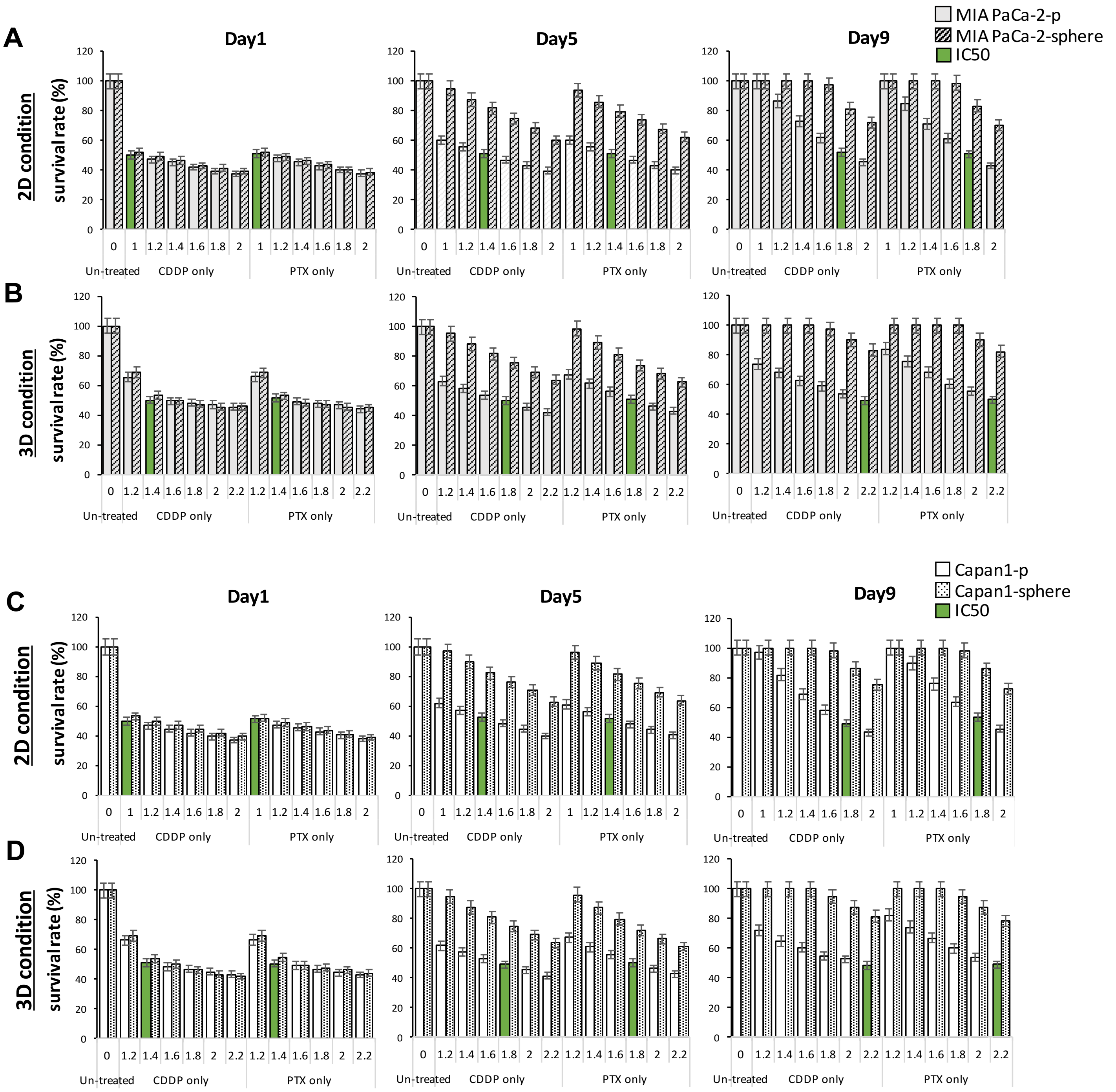

Figure S7A. Compare the growth inhibition of cisplatin (CDDP) and paclitaxel (PTX), respectively in MIA PaCa-2 parental cells (MIA PaCa-2-P) and MIA PaCa-2 induced-spheroid cells (MIA PaCa-2-sphere) derived from days 1, 5, and 9 pretreated with 1 μM CDDP + 1 nM PTX in 2D- and 1.4 μM CDDP + 1.4 nM PTX in 3D- culture conditions. Both CDDP and PTX were tested in the concentration ranges of 1.0-2.2 μM and 1.0-2.2 nM, respectively. The growth inhibition effect was considered significant when the inhibition rate was around 50% (IC50; green symbol). The IC50 of CDDP and PTX in A, 2D culture condition and B, 3D culture condition, respectively, are shown by indicated green color bars. Data shown were acquired from three independent experiments. **S7B. Compare the growth inhibition of cisplatin (CDDP) and paclitaxel (PTX), respectively, in Capan1 parental cells (Capan1-P) and Capan1 induced-spheroid cells (Capan1-sphere) derived from days 1, 5, and 9 pretreated with 1 μM CDDP + 1 nM PTX in 2D- and 1.4 μM CDDP + 1.4 nM PTX in 3D- culture conditions.** Both CDDP and PTX were tested in the concentration ranges of 1.0-2.2 μM and 1.0-2.2 nM, respectively. The growth inhibition effect was considered significant when the inhibition rate was around 50% (IC50; green symbol). The IC50 of CDDP and PTX in A, 2D culture condition and B, 3D culture condition, respectively, are shown by indicated green color bars. Data shown were acquired from three independent experiments.

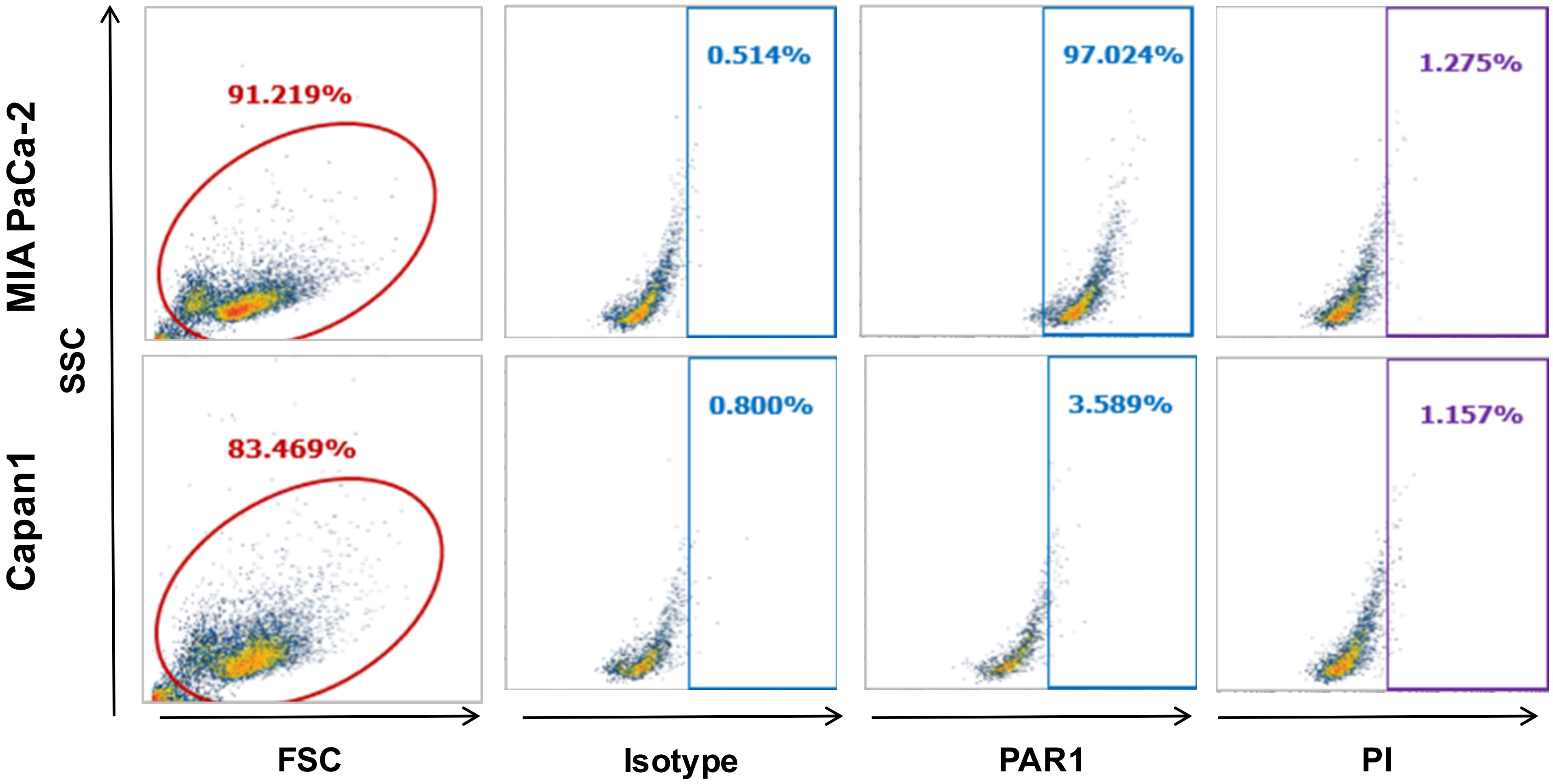

Figure S8. Endogenous PAR1 expression in human MIA PaCa-2 and Capan1 cells. PaC cell lines MIA PaCa-2 and Capan1 were cultured for PAR1 level screening. Flow cytometry analysis of PAR1 surface expression on both cell lines using PE-anti-PAR1 antibody versus an isotype control. PI staining was used for cell apoptosis detection. All data represent are at least from three independent experiments.
